# Carotid-cavernous fistula(CCF) presenting as paroxysmal painful ophthalmoplegia

**DOI:** 10.1186/s12886-019-1039-8

**Published:** 2019-02-11

**Authors:** Shan Li, Bin Feng, Yabo Feng, Zaiying Pang, Youting Lin

**Affiliations:** 1 Department of Neurology, Shandong Provincial Hospital affiliated to Shandong University, No 324, Jingwu Road, Huaiyin Zone, Jinan, Shandong Province 250012 People’s Republic of China; 2 Department of Neurosurgery, Shandong Provincial Hospital affiliated to Shandong University, Jinan, Shandong Province People’s Republic of China

## Abstract

**Background:**

Painful ophthalmoplegia can be caused by various etiologies, and broad differential diagnosis is needed. Carotid-cavernous fistula (CCF) is a rare cause of painful ophthalmoplegia, and early diagnosis is quite difficult.

**Case presentation:**

Here, we present a case of paroxysmal painful ophthalmoplegia caused by CCF. The episodic symptoms were nonstereotypical and lasted minutes to hours. Magnetic resonance imaging (MRI) and computed tomography angiography (CTA) results were normal, which confounded efforts to determine a diagnosis. Subsequently, digital subtraction angiography (DSA) revealed a posterior-draining CCF. The CCF was treated at an early stage without residual symptoms.

**Conclusions:**

We propose that symptoms could be relapsing or remitting during an early stage of CCF and that posterior-draining CCF is prone to misdiagnosis due to atypical manifestations. Normal CTA results cannot exclude carotid-cavernous fistula, and DSA should be performed once CCF is suspected.

## Background

The cavernous sinus (CS) is a venous plexus that receives drainage from the sphenoparietal sinus, the superior ophthalmic vein (SOV), the inferior ophthalmic vein (IOV), the superior petrosal sinus (SPS), the inferior petrosal sinus (IPS) and the basilar venous plexus. A carotid-cavernous fistula (CCF) consists of abnormal communications between the CS and branches of either the internal carotid artery (ICA) or the external carotid artery (ECA). [1] As a result, CCF can present with different symptoms, such as proptosis, blurred vision, chemosis, headache and ophthalmoplegia. [1] During an early stage, CCF may have atypical manifestations and is prone to being mistakenly identified as other pathologic cavernous sinus conditions. Here, we report a case of CCF with atypical manifestations and discuss major clinical characteristics of this case.

## Case presentation

A 60-year-old man was admitted for a 1-month history of paroxysmal left periorbital pain accompanied by various symptoms, including left ptosis, blurred vision in the left eye, and diplopia during each episode. Episode duration ranged from minutes to hours. The patient suffered from one to three attacks per day, and his condition continued to worsen. He had visited an oculist, and his visual acuity, visual field and intraocular pressure were normal. The patient had been diagnosed with hypertension 2 years prior and subsequently began taking extended-release nifedipine tablets. He denied any history of chronic headache, trauma or preliminary infection.

**Fig. 1:**
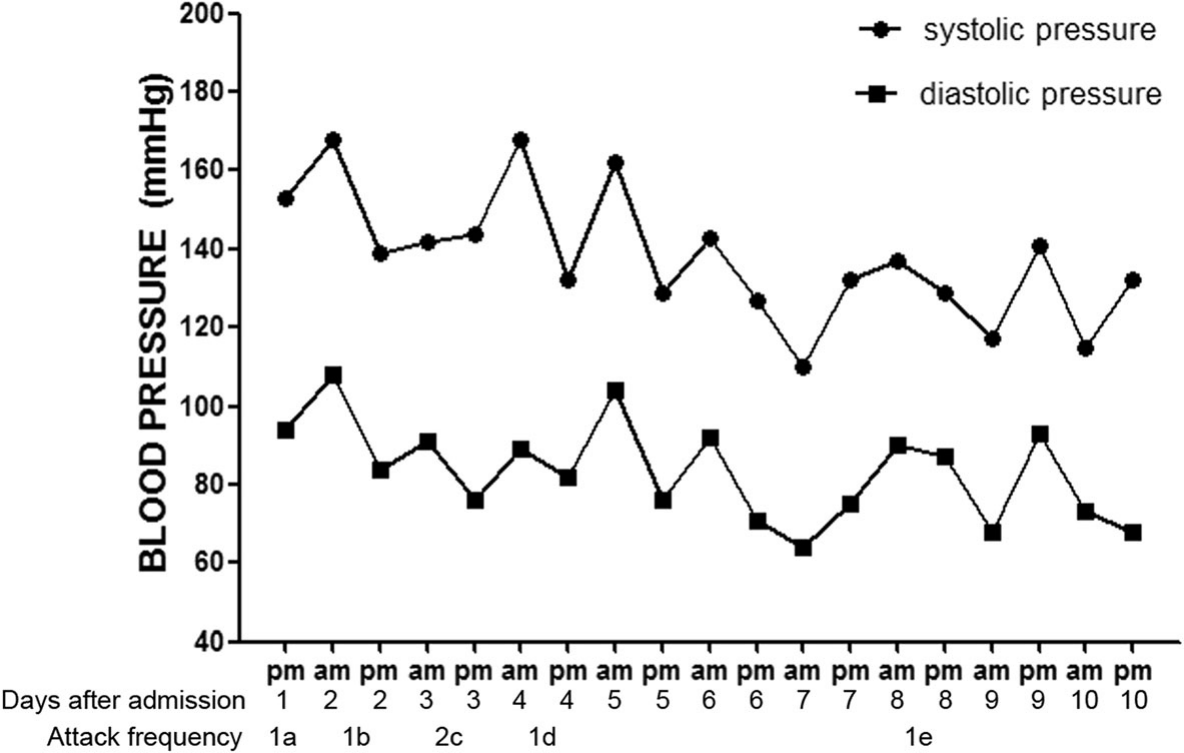
Blood pressure level and attacks during the first 10 days: **a**. periorbital pain for hours; **b**. periorbital pain accompanied by lachrymation and blurred vision; **c**. two attacks: 1) ptosis, mydriasis, diplopia, and slightly restricted supraduction, infraduction, and adduction for 10 min, 2) total oculomotor nerve paresis without pain for hours; **d**. ptosis and restricted adduction with a normal pupil for hours; **e**. periorbital pain and blurred vision for hours

At admission, neurologic examination produced unremarkable findings during symptom remission. By 7 days after admission, the patient had suffered 6 episodes, which are summarized in Fig. 1. The patient’s symptoms were due to impairment of different combinations of multiple cranial nerves (CNs), including the oculomotor nerve (CN3), the first division of the trigeminal nerve (CN 5–1) and the optic nerve (CN2), restricting the location of the lesion to the regions from the posterior cavernous sinus to the orbital apex.

Results for routine blood tests, erythrocyte sedimentation rate (ESR), rheumatoid factor and C-reactive protein were normal. Negative results were obtained for all tests for autoimmune antibodies and ultrasound assessments of temporal arteries. Lumbar puncture was performed with a pressure of 210 mmH_2_O, and tests revealed normal findings for cell counts, protein, and glucose as well as negative PCR results for herpes simplex virus type 1 and 2, cytomegalovirus, and EB virus. Computed tomography (CT) and contrast magnetic resonance imaging (MRI) revealed that brain structures, the orbital cavity, the cavernous sinus, and optic nerves were normal.

**Fig. 2:**
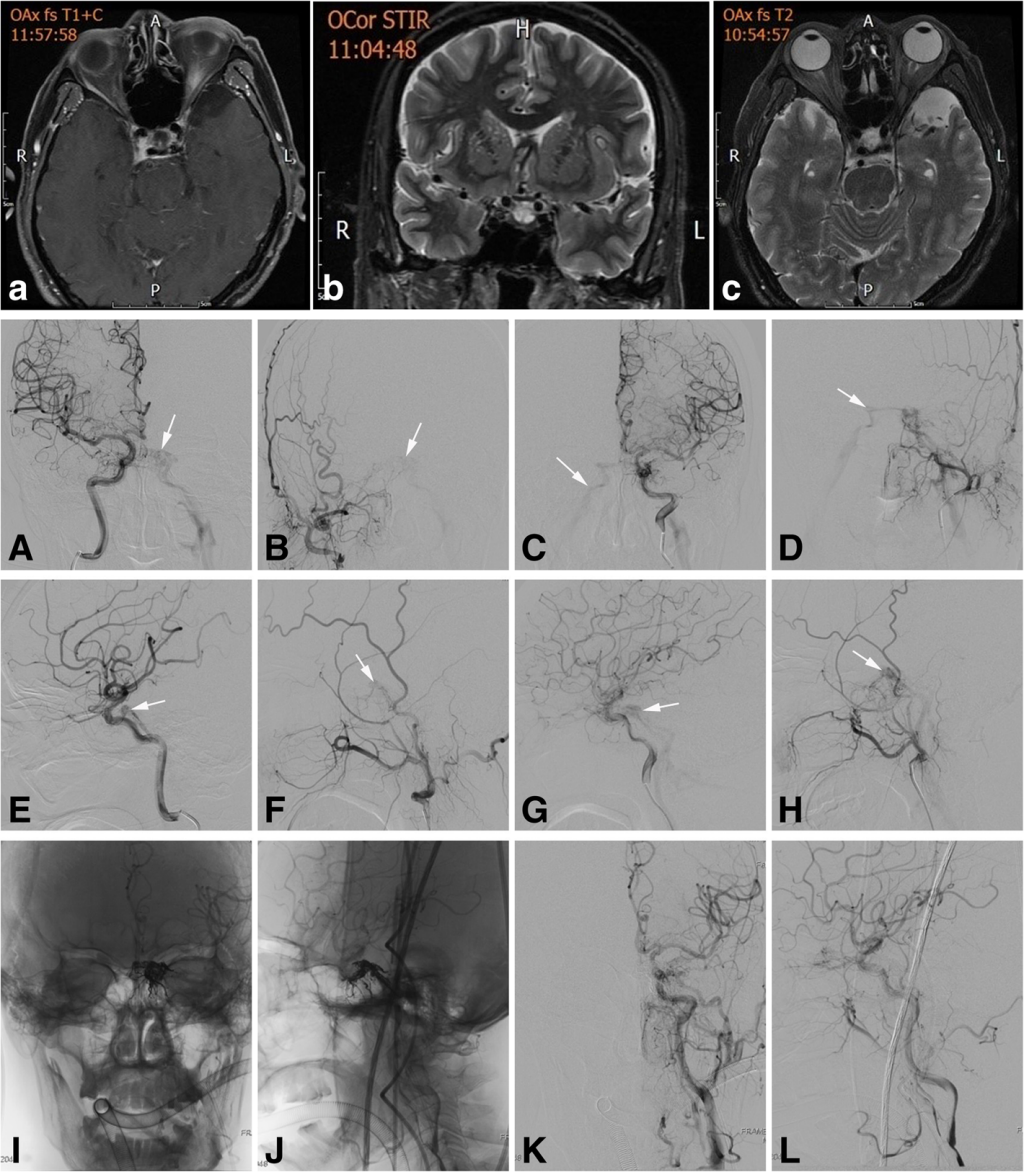
Enhanced T1-weighted (**a**) and T2-weighted (**b**,**c**) MRI showing normal cavernous sinuses. Early opacification of the bilateral cavernous sinuses: anteroposterior views of the right ICA (**a**) and ECA (**b**) and the left ICA (**c**) and ECA (**d**); lateral views of the right ICA (**e**) and ECA (**f**) and the left ICA (**g**) and ECA (**h**). Anteroposterior (**i**) and lateral (**j**) views showing the position of Onyx in the left cavernous sinus, the inferior petrosal sinus and the intercavernous sinus. Anteroposterior (**k**) and lateral (**l**) views of the left carotid confirmed complete occlusion of the CCF

Because the patient’s symptoms could disappear rapidly, even within minutes, angiopathy was considered. Computed tomography angiography (CTA) showed normal imaging of cervical and cerebral vessels and no tortuous vessels in the cavernous sinus. Transcranial Doppler ultrasonography (TCD) demonstrated an abnormal spectrum for the bilateral ophthalmic arteries (OA) with decreased PI and high flow velocity in the left OA. Ultimately, DSA confirmed bilateral CCF and shunts to the cavernous sinus from bilateral branches of the ICA and ECA (Fig. 2).

A microcatheter was passed through the left inferior petrosal sinus into the cavernous sinus, and Onyx, an embolic agent, was placed in the cavernous sinus for occlusion. Simultaneously, DSA was performed to ensure complete closure of the CCF without residual arteriovenous shunting (Fig. 2). The patient’s symptoms disappeared completely during more than 1 year of follow-up.

## Discussion

CCFs are abnormal vascular shunts that allow blood to either directly or indirectly flow from the carotid artery into the CS. An anatomical classification defines direct and indirect CCFs as those arising directly from the carotid artery and those originating from carotid artery branch vessels, respectively. [1] Compared with direct CCFs, indirect CCFs are usually spontaneous and infrequently traumatic. They are more common in the elderly [2, 3] and may be associated with predisposing factors such as hypertension, atherosclerosis, or connective tissue diseases. [4, 5]

In this case, the disease course was relapsing and remitting in nature, and the manifestations were diverse rather than stereotypical. These features are rare for CCF and merit consideration. It was observed that to an extent, episode onset was associated with the patient’s blood pressure level. As proposed by Ellis et al., the precise pattern of symptoms was dependent on the location of venous drainage of the CCF and the pressure within the venous sinus [1]. We presumed that hypertension could lead to gradual hyperperfusion in the CS that could be reversible during an early stage of CCF. To varying degrees, elevated blood pressure was accompanied by the CS being influenced to different extents, and the corresponding manifestations lasted for shorter or longer durations. Thus, early diagnosis is crucial to prevent progression.

Obvious proptosis, chemosis and orbital bruits are typical signs pointing to CCF [6, 7]; however, symptoms for CCF differ according to the distribution of venous drainage. These congestive manifestations are associated with anterior-draining CCF, which influences the SOV and is called “red eye shunt”, [3] whereas isolated ocular motor nerve palsy without congestive ocular features is correlated with posterior-draining CCF, which influences mainly the SPS and IPS and is called “white eye shunt”. [3] According to DSA results for our patient, the lesion location was confined mainly within the posterior cavernous sinus, which explains why typical congestive manifestations were absent.

In fact, posterior-draining CCF is seldom first considered and is prone to be mistaken as Tolosa-Hunt syndrome (THS) or aneurysm. In most cases, once ophthalmoplegia has occurred, symptoms will persist for days to weeks, and painful ophthalmoplegia that remits and relapses within a day is extremely rare. [8] Similar statements are applicable for recurrent painful ophthalmoplegic neuropathy (RPON), which has been accepted as a chronic inflammatory painful neuropathy. [7] Episode duration was much shorter in this case than in most cases of these inflammatory neuropathies. Oculomotor palsy in addition to pupillary involvement is highly suggestive of an intracranial aneurysm, which was excluded via CTA in this case. Differential diagnosis of episodic painful ophthalmoplegia involving CN2/3/5–1 should include other pathologic conditions in the cavernous sinus or orbital apex, such as malignancies, inflammation or infections; in this case, such conditions were excluded based on the normal CSF and neuroimaging results.

Signs that can be detected using noninvasive imaging include proptosis, dilatation of the SOV, dilatation of cortical or leptomeningeal vessels, extraocular muscle enlargement, ipsilateral CS enlargement, and skull fracture. [1, 2] However, normal CTA and MR imaging cannot exclude CCF, [9] especially in cases involving indirect CCFs with low flow velocity. DSA is the gold standard and should be performed in cases in which CCF is strongly suspected.

## Conclusion

We propose that at an early stage of CCF, the disease course could be relapsing and remitting in nature, particularly for elderly patients with unstable predisposing factors such as hypertension. Posterior-draining CCF is prone to be misdiagnosed because of a lack of typical congestive manifestations. Negative CTA results cannot exclude CCF, and DSA should be performed once CCF is suspected. CCF is treatable, and early diagnosis is important for prognosis.

## Abbreviation

CCF: Carotid-cavernous fistula
CN: Cranial nerves.
CS: Cavernous sinus.
CTA: Computer Tomography Angiography.
DSA: Digital subtraction angiography.
ECA: Externalcarotid artery.
ESR: Erythrocyte sedimentation rate.
ICA: Internal carotid artery.
IOV: Inferior ophthalmic vein.
IPS: Inferior petrosal sinus.
MRI: Magnetic Resonance Imaging.
OA: Ophthalmic artery.
RPON: Recurrent painful ophthalmoplegic neuropathy.
SOV: Superior ophthalmic vein.
SPS: Superior petrosal sinus.
TCD: Transcranial Doppler ultrasonography.
THS: Tolosa-Hunt syndrome.
